# Alteration in glucocorticoids secretion and metabolism in patients affected by cystic fibrosis

**DOI:** 10.3389/fendo.2022.1074209

**Published:** 2022-12-08

**Authors:** Rafał Podgórski, Marta Sumińska, Marta Rachel, Marta Fichna, Piotr Fichna, Artur Mazur

**Affiliations:** ^1^ Department of Biochemistry, Institute of Medical Sciences, Medical College of Rzeszow University, Rzeszow, Poland; ^2^ Department of Pediatric Diabetes, Auxology and Obesity, Institute of Pediatrics, Poznan, University of Medical Sciences, Poznan, Poland; ^3^ Department of Pediatrics, Institute of Medical Sciences, Medical College of Rzeszow University, Rzeszow, Poland; ^4^ Department of Endocrinology, Metabolism and Internal Medicine, Poznan University of Medical Sciences, Poznan, Poland

**Keywords:** cystic fibrosis, cortisol metabolites, 11β-hydroxysteroid dehydrogenase activity, 5α-reductase activity, 11βHSD1

## Abstract

Cystic fibrosis (CF) is an inherited syndrome associated with a mutation in a cystic fibrosis transmembrane conductance regulator gene, composed of exocrine gland dysfunction involving multiple systems that may result in chronic respiratory infections, pancreatic enzyme deficiency, and developmental disorders. Our study describes for the first time the urinary profile of glucocorticoid metabolites and the activity of the enzymes involved in the development and metabolism of cortisol in patients with CF, using a gas chromatography/mass spectrometry method. Data were obtained from 25 affected patients and 70 sex- and age- matched healthy volunteers. We have shown a general decrease in the activity of enzymes involved in the peripheral metabolism of cortisol, such as 11β-hydroxysteroid dehydrogenase type 2, 5α- and 5β-reductases. In contrast, the activity of 11β-hydroxysteroid dehydrogenase type 1, the enzyme that converts cortisone to cortisol, increased. Furthermore, our study found a significant decrease in glucocorticoid excretion in patients with CF. This may suggest adrenal insufficiency or dysregulation of the HPA axis and the development of peripheral mechanisms to counteract cortisol degradation in the case of reduced synthesis of glucocorticoids by the adrenal glands. Furthermore, the activity of 5α-reductase seems to be enhanced only through the backdoor pathway, especially when we taking into consideration 11β-hydroxyandrosterone/11β-hydroxyetiocholanolone ratio which has been shown to be the best differential marker for enzyme activity. CF impairs nutritional effects and energetic balance in patients; thus, our findings suggest the existence of adaptive mechanisms due to limited secretion of adrenal steroids and subsequent diminished amounts of their metabolites in urine. On the other hand, local control of cortisol availability is maintained by enhanced 11βHSD1 activity and its recovery from cortisone in organs and tissues which need this. Steroid hormone dysregulation might be another important factor in the course of CF that should be taken into account when planning an effective and comprehensive therapy.

## Introduction

Cystic fibrosis (CF) is a life-limiting autosomal recessive inherited monogenic disorder with the highest prevalence in the Caucasians populations with a mean incidence ranging from 1:1353 in Ireland and 1:4394 in Poland to 1:10 000 in Russia and in African-Americans (1, 2). CF is caused by a mutation in a gene that encodes a chloride-conducting transmembrane channel termed the cystic fibrosis transmembrane conductance regulator (CFTR), located in the long arm of chromosome 7 (7q31) (3). CFTR regulates chloride anion transport and mucociliary clearance predominantly in the airways. *CFTR* is also expressed in other tissues, where it regulates both secretion and absorption, including the pancreatic duct epithelium, the crypts of the small intestinal epithelium, the liver, sweat glands, and the reproductive tract (4, 5). More than 2000 different variants of the *CFTR* gene sequence have been found, and 20% of them are established as pathogenic (6). CFTR dysfunction causes chronic obstructive pulmonary disease, hyperresponsiveness in asthma, disturbances of the digestive system with exocrine pancreatic insufficiency, and fertility problems in both sexes (7). The regulation of *CFTR* expression is complex, and many mechanisms are involved in producing the tissue-specific regulation exhibited by this gene. One of the *CFTR* regulating mechanisms is hormonal control. In rats, estrogens have been found to increase CFTR activity in the uterine epithelium of the oviductal mucosa (8). There are also glucocorticoid response elements in the *CFTR* promoter, and glucocorticoid treatment inhibits *CFTR* expression and activity (9). Despite the demonstration of hormonal regulation of *CFTR* expression, the regulatory elements responsible have not been clearly recognized. Adrenal steroid hormones are produced in a very complex cycle that is regulated by many factors, including the hypothalamic-pituitary-adrenal (HPA) axis. It requires the interaction of a cascade of enzymes that result in their formation as well as other enzymes for their further metabolism.

**Figure 1:**
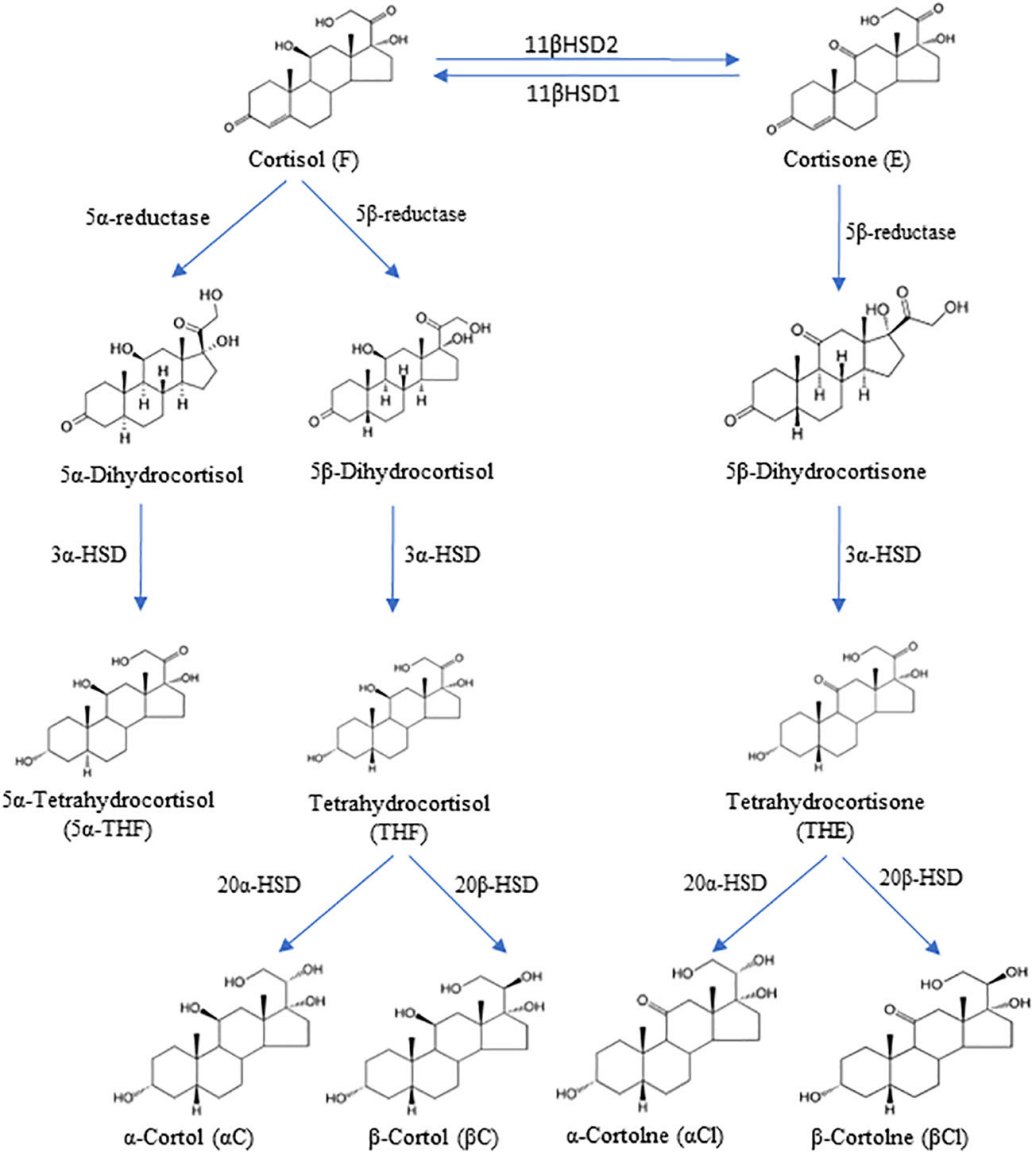
The degradation pathway of cortisol and cortisone and their metabolites. 11β-hydroxysteroid dehydrogenase type 1 (11βHSD1), 11β-hydroxysteroid dehydrogenase type 2 (11βHSD2), 3α-hydroxysteroid dehydrogenase **(**3αHSD), 20α-hydroxysteroid dehydrogenase (20αHSD), 20β-hydroxysteroid dehydrogenase (20βHSD).

The main representative of glucocorticoids is cortisol, which contributes to the multifaceted regulation of the human body and the preparation of an appropriate response to stress. Cortisol stimulates gluconeogenesis in the liver, proteolytic processes in muscle, and lipolysis in adipose tissues, and increases plasma glucose concentrations to provide energy in starvation or for the stress response. In addition, cortisol affects the immune system response, the sleep process, as well as basic functions, such as growth and reproduction (10). Several enzymes are involved in the peripheral metabolism of cortisol, such as two distinct isozymes of 11β-hydroxysteroid dehydrogenase (11βHSD), 5α-reductase (SRD5A) and 5β-reductase (SRD5B), 3α-hydroxysteroid dehydrogenase (3αHSD) and 20α-hydroxysteroid dehydrogenase (20αHSD) or 20β-hydroxysteroid dehydrogenase (20βHSD) (**Figure 1**).

CF is not known to directly affect the adrenal gland, but commonly used CF therapies may have an impact on HPA function. By binding to the glucocorticoid receptor, drugs such as inhaled and oral corticosteroids can enhance the systemic effects of cortisol and even cause iatrogenic Cushing syndrome. On the other hand, long-term use of steroids inhibits the body’s ability to produce cortisol, causing iatrogenic adrenal insufficiency after discontinuation of such a systemic medication. Prolonged use of inhaled and mainly oral corticosteroids can adversely affect bone health, growth, and glucose metabolism (11). However, steroids are not commonly used as a leading treatment of CF (12, 13).

When assessing the impact of the disease on the endocrine system, we must remember that CF is a systemic disorder with particular manifestation in certain organs such as the liver, which are also involved in peripheral distribution and metabolism of steroid hormones. Impairment of the respiratory system is linked to inadequate oxygen access for energetic metabolism and can cause chronic stress conditions. Affecting such vital organs by this disease must induce many adaptive processes in the body to limit undesirable effects. The endocrine system, including the HPA axis, plays a significant role, with its *ad hoc* actions in stressful situations, as well as by long-term and rhythmic effects on metabolism in essential areas involving carbohydrates, proteins, and lipids. This axis can be expected to reveal adaptive responses in terms of secretory activity or metabolism of already circulating steroids. Examination of the steroid metabolome by analyzing the diurnal profiles of steroids excreted in urine is a very useful tool for a comprehensive and simultaneous assessment of such changes. Gas chromatography-mass spectrometry (GC-MS) is a powerful discovery tool for defining steroid disorder metabolomes. Since the 1930s, almost all inborn errors in steroidogenesis have been first defined through their urinary steroid excretion. In the last 30 years, this has been carried out by GC-MS and has defined conditions such as glucocorticoid remediable aldosteronism, apparent mineralocorticoid excess syndrome, Smith-Lemli-Opitz syndrome, P450 oxidoreductase deficiency, or apparent cortisone reductase deficiency. Using the single run of GC-MS technique allows to analyse every steroid excreted and provide an integrated picture of an individual’s metabolome (14).

The purpose of this study was to assess the impact of a serious systemic disease such as CF on adrenal glucocorticoid secretion and the activity of key enzymes involved in steroidogenesis and metabolism of cortisol in patients with CF. The early clinical manifestation of CF in children usually indicates on more severe course of this disease, thus we can expect the more evident changes in subject of our study. Moreover, to the best of our knowledge, this is the first paper to describe these aspects.

## Materials and methods

### Subjects

The study comprised 25 affected participants (13 girls, 12 boys) and 70 healthy control (32 girls, 38 boys), aged between 5 and 18 years. 24h urine collection was conducted according to the standard protocol. The study was performed in accordance with the Declaration of Helsinki and approved by the Bioethical Commission of the University of Rzeszow (Decision number 3/12/2018). Informed consent was obtained from patients at least 16 years of age and, in the case of minors, from their legal representatives. The diagnosis of CF diagnosis was confirmed based on the determination of sweat chloride, genetics, and the immunoreactive trypsin test in neonatal age (patients born in or after 2009). The next criteria for enrolling patients in the study were the following: forced expiratory volume in the first second (FEV_1_) greater than 35% of predicted stable pulmonary disease, defined by both clinical examinations and no hospitalizations in the 30 days prior to screening. Exclusion criteria were also as follows: heart and liver failure, psychiatric disorder, post-solid organ transplantation, and corticosteroids treatment. All CF patients suffered from pancreatic insufficiency and received pancreatic enzyme replacement therapy (Creon 25000, Solvay Pharmaceutical Inc., Marietta, Georgia, USA). The patients were also treated with human DNase I recombinant (Pulmozyme, Genentech Inc., San Francisco, California, USA; one 2.5 mg ampoule inhaled once a day using a nebulizer), fat-soluble vitamins in the form of ADEK tablets (Scandipharm, Birmingham, Alabama, USA), supplemental nutrition drinks (Nutrison Protein Plus, Nutricia, Poland) and inhalation of 3–10% sodium chloride 3-4 times a day.

Healthy subjects aged 5-17 years were sex-matched volunteers with no diseases in medical history or physical examination. The volunteers had not taken any medications 30 days before the study. All participants had anthropometric measurements. The body mass index (BMI) was calculated as kg/m^2^ and body surface area (BSA) as m^2^ according to Mosteller formula (square root of (height (cm) x weight (kg)/3600)).

### Quantification of urinary steroid metabolites

24-hour urine samples collection was performed at home for control and during outpatient visit for CF patients, according to the written collection procedure. Samples were stored at -20°C and analyzed by the in-house adapted GC-MS method as previously described (15–18). Briefly, the method involves initial extraction of a urine sample on a Sep-Pak C18 column, then enzymatic hydrolysis, re-extraction on a Sep-Pak cartridge, derivatization of the unconjugated steroids and after adding the internal standards purification on a Lipidex 5000 column. Finally, the dried residue was resuspended in cyclohexane and analyzed by Shimadzu 2010 Plus gas chromatograph coupled with Shimadzu QP-2010 Ultra mass spectrometer in selected ion monitoring mode (SIM).

Detailed description of the methodology used for the urinary steroid quantification is presented in **Supplementary File 1**.

### Steroid enzyme activities assessment

Metabolite ratio analysis was performed to assess steroid enzyme activity in accordance with previous publications on the diagnosis of various forms of adrenal dysfunction (16, 18–20). In detail, the ratio of the main tetrahydrometabolites of cortisol (THF plus 5αTHF) to those of cortisone (THE) provides a reflection of 11β-hydroxysteroid dehydrogenase type 1 activity.

To assess 11β-hydroxysteroid dehydrogenase type 2 activity the ratio of urinary free cortisone (E) to cortisol (F) as well as cortolones (αCl plus βCl) to cortols (αC plus βC) were calculated.

The activity of 5α-reductase, located mainly in the liver, can be determined by the ratios of 5α-dihydrotestosterone (5α-DHT) to testosterone (T) and androsterone (An) to etiocholanolone (Et), 5α-tetrahydrocorticosterone (5αTHB) to tetrahydrocorticosterone (THB), and 5a-tetrahydrocortisol (5αTHF) to tetrahydrocortisol (THF). We have also hypothesized that the 11β-hydroxyandrosterone (11β-OH-An) to 11β-hydroxyetiocholanolone (11β-OH-Et) ratio will reflect the 5α-reductase activity and – indirectly – the androgen metabolism after their second crossing through the adrenal cortex.

5β-reductase is involved in the metabolism of cortisol and cortisone in cooperation with 3α-hydroxysteroid dehydrogenase (3αHSD) due to the activity of the 5β-reductase+3αHSD complex was evaluated using the ratios [α-cortol (αC)+β-cortol (βC)+THF]/F and [α-cortolone (αCl)+β-cortolone (βCl)+THE]/E.

The activity of 20αHSD was assessed by the ratio of [αC+αCl]/[THF+5αTHF+THE] and the activity of 20βHSD by the [βC+βCl]/[THF+5αTHF+THE] ratio.

The sum of 7 main glucocorticoid metabolites C21 (THE, THF, 5αTHF, αC, βC, αCl and βCl) and all measured cortisol metabolites (F, E, THF, 5αTHF, THE, αC, βC, αCl, βCl, 20α-DHF, 20α-DHE, 20β-DHE, 20β-DHF, 6β-OH-F) provide a reflection of preliminary substrates load of glucocorticoid secretion rate.

### Statistical analyses

The ratios of the 24h urinary excretion of steroid metabolites were compared using Statistica 13 (Dell Inc., Tulsa, OK, USA). Steroid excretion rates were normalized to body surface area (BSA), because of a close functional and anatomical correlation between adrenal volume and BSA (21–24). Most variables did not follow a normal distribution, which was assessed using the Shapiro-Wilk W test; therefore, the Mann-Whitney U test was used for comparisons between patients with CF and controls. The correlation analysis was performed using the Spearman correlation test. A p-value less than 0.05 was considered statistically significant. Data are presented as median and mean ± SD. The accuracy of different classifier used to determine the steroid enzyme activities to differentiate patients into CF and healthy was evaluated by the area under the curve (AUC) and its 95% CI of a receiver operating characteristic (ROC) analysis using the MedCalc software version 20.111 (Ostend, Belgium).

## Results

95 participants were enrolled into the study and 25 patients (26,3%) were affected by CF. The mean values ± SD for basic anthropometric parameters, BSA, clinical laboratory markers and pulmonary function are presented in **Table 1**. The mean age, height, weight, BMI, BMI Z-Score, as well as BSA were not significantly different between the participants. Biochemical markers were determined only in the CF group during treatment.

**Table 1:** Baseline demographic and clinical data of the study participants.

		CF	Healthy controls	*p*
Sex (F/M)		13/12	32/38	
Age (years)	mean ± SD	12.24 ± 3.62	10.813 ± 3.16	0.084
Height (cm)	mean ± SD	151.88 ± 17.52	146.95 ± 20.90	0.326
Height Z-Score	mean ± SD	-0.31 ± 1.15	-0.02 ± 1.02	0.329
Weight (kg)	mean ± SD	44.24 ± 15.70	43.70 ± 18.55	0.604
Weight Z-Score	mean ± SD	0.20 ± 1.20	-0.06 ± 1.00	0.672
BMI (kg/m^2^)	mean ± SD	18.67 ± 3.55	17.98 ± 2.99	0.427
BMI Z-Score	mean ± SD	-0.17 ± 0.97	-0.01 ± 1.04	0.310
BSA (m^2^)	mean ± SD	1.35 ± 0.32	1.26 ± 0.37	0.269
Genotype				
Homozygous ΔF508. n (%)		20 (80.0%)	–	–
Heterozygous ΔF508. n (%)		5 (20.0)	–	–
Clinical laboratory markers				
CRP (mg/L)	mean ± SD	5.54 ± 6.1	–	**-**
IL-6 (pg/ml) Norm <7	mean ± SD	6.19 ± 10.35		–
NEU (%)	mean ± SD	61.01 ± 15.3	–	**-**
WBC (10^3^/µL)	mean ± SD	9.95 ± 3.6	–	**-**
Cholesterol (mg/dL)	mean ± SD	117.67 ± 17.43	–	–
HDL (mg/dL)	mean ± SD	42.89 ± 9.73	–	–
LDL (mg/dL)	mean ± SD	66.89 ± 16.53	–	–
Triglycerides (mg/dL)	mean ± SD	84.7 ± 43.5	–	–
Pulmonary function				
FEV_1_ (L)	mean ± SD	2.39 ± 0.87	–	**-**

BMI, body mass index; BSA, body surface area; IL-6, Interleukin 6; WBC, white blood cells.

NEU, neutrophils; CRP, C-reactive protein; FEV1, forced expiratory volume in 1 second; data are presented as mean ± SD; differences between means were analyzed using Mann-Whitney U test.

In **Table 2** are presented 24-h urinary excretion rates of the most important single and aggregated glucocorticoid metabolites, corrected for BSA. The BSA correction was calculated as: excretion of each steroid metabolites (μg/24h) divided by BSA (m^2^) of the subject. **Table 2** also contains the comparison excretion of steroid metabolites between CF and controls using the Mann-Whitney U test. All compounds, with the exception of Et and 20α-DHF have shown a significant difference in the excretion of metabolites. Generally, CF patients reveled a lower excretion rate of the main glucocorticoid metabolites. The largest decrease in the median excretion of glucocorticoid metabolites was observed in the CF for 20β-DHF (3.5- fold, p<0.001), βCl and αC (almost 2.5-fold, p <0.001). A significantly lower content of the sum (Σ) of the main glucocorticoids metabolites was also observed for the C21 metabolites (1.9- fold, p<0.001) and all cortisol metabolites (1.8-fold, p<0.001).

**Table 2:** Urinary 24-h excretion rates of steroid metabolites (body surface area corrected).

Steroid hormone/(Abbreviation)[μg/24h]	CF patients		Control patients		
	Mean ± SD	Median (IQR)	Mean ± SD	Median	p
Androsterone (An)	455.42 ± 505.32	239.24 (118.71-638.47)	803.17 ± 828.91	478.23 (190.31-1218.59)	**0.047**
Etiocholanolone (Et)	276.52 ± 286.13	163.53 (62.74-429.79)	497.48 ± 612.39	302.20 (99.39-595.29)	0.121
11β-Hydroxyandrosterone (11β-OH-An)	311.45 ± 279.15	257.49 (117.78-383.39)	582.30 ± 437.38	487.88 (283.75-753.63)	**<0.001**
11β-Hydroxyetiocholanolone (11β-OH-Et)	13.70 ± 11.26	11.21 (7.28-17.85)	161.93 ± 229.98	109.46 (35.65-188.61)	**<0.001**
Tetrahydrocortisone (THE)	1040.50 ± 795.80	900.64 (401.46-1352.92)	1943.31 ± 827.12	1791.23 (1386.05-2354.22)	**<0.001**
Tetrahydrocortisol (THF)	460.03 ± 338.18	273.89 (196.57-643.63)	599.48 ± 300.18	519.95 (406.45-763.69)	**0.025**
5a-Tetrahydrocortisol (5α-THF)	521.94 ± 431.75	332.52 (179.86-852.98)	871.65 ± 478.20	759.64 (529.97-1119.73)	**0.001**
α -Cortolone (αCl)	394.02 ± 469.28	314.25 (154.43-457.83)	862.94 ± 488.77	737.70 (497.38-1083.37)	**<0.001**
β-Cortolone (βCl)	161.62 ± 130.45	124.03 (68.15-210.97)	332.02 ± 148.10	303.52 (245.67-383.51)	**<0.001**
α -Cortol (αC)	104.13 ± 90.93	94.50 (36.98-130.92)	157.11 ± 77.33	144.88 (91.97-216.35)	**0.001**
β-Cortol (βC)	151.89 ± 112.82	133.98 (97.85-183.26)	255.49 ± 124.60	224.38 (165.15-319.80)	**<0.001**
Cortisone (E)	61.30 ± 46.62	50.24 (26.62-86.41)	87.77 ± 36.11	80.85 (60.56-110.68)	**0.002**
Cortisol (F)	56.76 ± 46.71	41.09 (18.24-80.38)	94.43 ± 41.42	81.62 (61.05-118.07)	**<0.001**
20β-Dihydrocortisone (20β-DHE)	24.25 ± 33.05	15.18 (6.9-29.39)	40.16 ± 20.92	33.73 (23.49-57.69)	**<0.001**
20α-Dihydrocortisone (20α-DHE)	10.40 ± 8.50	7.08 (4.72-12.58)	14.62 ± 7.18	13.71 (9.06-18.45)	**0.003**
20β-Dihydrocortisol (20β-DHF)	67.94 ± 68.77	38.13 (27.54-71.69)	156.99 ± 121.80	134.69 (65.81-210.99)	**<0.001**
6β-hydroxycortisol (6β-OH-F)	29.24 ± 28.43	15.54 (11.34-40.58)	33.58 ± 16.99	28.59 (21.07-44.4)	**0.018**
20α -Dihydrocortisol (20α-DHF)	47.67 ± 59.40	18.39 (8.39-57.61)	26.52 ± 17.69	21.23 (12.52-33.9)	0.963
THF+5αTHF+THE	2022.47 ± 1493.01	1790.91 (748.55-2828.33)	3414.44 ± 1361.52	3323.99 (2441.3-3814.49)	**<0.001**
Σ C21 metabolites	2834.13 ± 2162.25	2590.10 (1062.72-3865.36)	5022.01 ± 1953.97	4854.88 (3643.91-6151.48)	**<0.001**
Σ Cortisol metabolites	3131.70 ± 2331.35	2903.26 (1178.5-4252.46)	5476.08 ± 2076.08	5165.59 (4031.89-6695.68)	**<0.001**

IQR, interquartile range, Σ Cortisol metabolites- the sum of: F, E, THF, 5αTHF, THE, αC, βC, αCl, βCl, 20α-DHF, 20α-DHE, 20β-DHE, 20β-DHF, 6β-OH-F; Σ C21 metabolites- the sum of: THE, THF, 5αTHF, αC, βC, αCl and βCl. Statistically significant values were bolded.

To assess the activity of key enzymes involved in the metabolism of glucocorticoids, typical product to substrate ratios were calculated and compared between affected and healthy children. Comparison between enzyme activities in the study groups was performed using the Mann-Whitney U test and has shown statistically significant differences in the activities of many enzymes involved in steroid metabolism (**Table 3**). The conversion product to substrate ratio of steroid metabolites reflecting the 11βHSD1 activity ((THF+5αTHF)/(THE), 0.98 ± 0.32 vs. 0.80 ± 0.28, p=0,013) was higher for CF, indicating a significant increase in the activity of this enzyme in CF compared to the control. To assess the activity of 11β-HSD2 two different ratios of steroid metabolites were evaluated and revealed statistically significant changes in 11β-HSD2 activity: (E/F, 1.15 ± 0.45 vs. 0.797 ± 0.28, p=0.009 and cortolones/cortols, 2.21 ± 0.92 vs. 2.98 ± 0.94; p=0.001), which seemed to be lower in CF. The comparison ratios used to assess SRD5A activity have shown conflicting results. The activity of SRD5A measured by the 5α-DHT/T, 5αTHB/THB, 5αTHF/THF and An/Et ratios showed a lower conversion ability of the enzyme studied. On the contrary, calculations of the 11β-OH-An/11β-OH-Et ratio revealed an increase in SRD5A activity in CF. These findings were not statistically significant only in the case of the An/Et ratio. Analysis of (αC+ βC+THF)/F and (αCl+ βCl+THE]/E) ratios, reflecting the activity of the enzyme complex SRD5B + 3αHSD, gave contradictory results, but both calculated ratios were statistically significant. Statistical analyses did not reveal significant differences in the activity of the 20αHSD and 20βHSD activity.

**Table 3:** Enzymes activity calculated from the product/substrate ratios and comparison between CF children and adolescents and healthy control.

Product/substrate ratio	CF		Control		CF
	Mean ± SD	Median (IQR)	Mean ± SD	Median (IQR)	p
**11β-hydroxysteroid dehydrogenase type 1 activity**					
(THF+5αTHF)/(THE)	0.98 ± 0.32	0.99 (0.76-1.11)	0.80 ± 0.28	0.74 (0.6-0.99)	**0.013**
**11β-hydroxysteroid dehydrogenase type 2 activity**					
E/F	1.15 ± 0.45	1.04 (0.97-1.27)	0.96 ± 0.20	0.94 (0.79- 1.12)	**0.009**
Cortolones/cortols	2.21 ± 0.92	2.12 (1.50-2.59)	2.98 ± 0.94	2.88 (2.23-3.51)	**0.001**
**5α- reductase activity**					
An/Et	1.80 ± 0.73	1.60 (1.32-2.09)	1.93 ± 0.82	1.86 (1.30-2.49)	0.574
11β-OH-An/11β-OH-Et	28.16 ± 18.64	24.01 (14.23-38.46)	11.11 ± 20.63	4.18 (2.37-8.61)	**<0.001**
5α-DHT/T	0.55 ± 0.47	0.28 (0.17-0.97)	0.78 ± 0.62	0.60 (0.36-0.95)	**0.031**
5αTHB/THB	3.33 ± 1.87	2.97 (2.08-4.00)	4.41 ± 2.06	4.30 (3.03-5.26)	**0.005**
5αTHF/THF	1.14 ± 0.44	1.03 (0.85-1.47)	1.60 ± 0.73	1.47 (1.08-2.09)	**0.004**
**5β- reductase + 3α-hydroxysteroid dehydrogenase activity**					
[αC+ βC+THF]/F	13.85 ± 5.13	12.44 (10.69-16.36)	11.19 ± 3.89	10.59 (9.04-12.93)	**0.006**
[αCl+ βCl+THE]/E	29.61 ± 4.92	26.40 (19.42-33.26)	37.52 ± 12.78	35.34 (28.37-40.87)	**0.001**
**20α-hydroxysteroid dehydrogenase activity**					
[αC+αCl]/[THF+5αTHF+THE]	0.25 ± 0.10	0.24 (0.18-0.30)	0.30 ± 0.11	0.27 (0.22-0.36)	0.077
**20β-hydroxysteroid dehydrogenase activity**					
[βC+βCl]/[THF+5αTHF+THE]	0.16 ± 0.05	0.15 (0.13-0.2)	0.18± 0.06	0.17 (0.14-0.21)	0.277

Please refer to **Table 2**. Statistically significant values were bolded.

**Figure 2:**
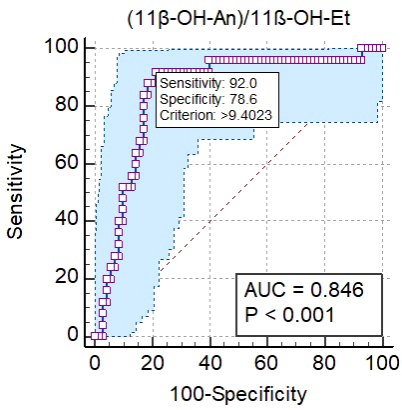
Results of the analysis of the ROC curve for 11β-OH-An/11β-OH-Et ratio. The blue shaped area is the 95% confidence interval of the sensitivity at the given specificity.

The performance of urinary steroid metabolite ratios was also evaluated to identify the best distinguishing factor to differentiate CF from controls. The best ratio was 11β-OH-An/11β-OH-Et with AUC 0,846 (95% CI: 0,754 to 0,909) under the ROC curve, which yields a specificity of 78.6% and a sensitivity of 92.0% to distinguish alterations in steroid enzyme activities in CF patients from controls (**Figure 2**).

Detailed results of the analysis are presented in **Supplementary File 2** (**Figures S1**- **S12**).

The correlation analysis of the steroid metabolite ratios and the clinical parameters of the studied patients has shown a moderate association of (THF+5αTHF)/THE (R= 0.513, p= 0.009), 5α-DHT/T (R= -0.507, p= 0.010), cortolones/cortols (R= -0.497, p= 0.012), (R= -0.432 p= 0.031) and 5αTHB/THB (R= -0.410, p= 0.042) with age. Taking into account the BMI ratios of [αC+ βC+THF]/F (R= 0.413, p= 0.040), (THF+5αTHF)/THE (R= 0.449, p= 0.024) are correlated positively, cortolones/cortols (R= -0.497, p= 0.012) and 5α-DHT/T (R= -0.481, p= 0.015) negatively. BSA is moderately correlated with the cortolones/cortols ratio (R= -0.482, p= 0.015), [αCl+ βCl+THE]/E (R= -0.423, p= 0.035) (THF+5αTHF)/THE (R= 0.594, p= 0.002). Inflammation markers such as CRP and IL-6, pulmonary function (FEV1) and BMI Z-Score are not correlated with any of the parameters studied. Detailed results are presented in **Table 4**.

**Table 4:** Spearman’s rank correlation coefficients and p values in CF patients.

		Age	BMI	BSA	CRP	FEV_1_
(THF+5α-THF)/THE	*R*	**0.513**	**0.449**	**0.594**	0.293	0.393
	*p*	**0.009**	**0.024**	**0.002**	0.175	0.063
Cortolones/cortols	*R*	**-0.432**	**-0.497**	**-0.482**	-0.118	-0.294
	*p*	**0.031**	**0.012**	**0.015**	0.593	0.174
5α-THB/THB	*R*	**-0.410**	-0.334	-0.246	-0.313	-0.112
	*p*	**0.042**	0.103	0.236	0.145	0.610
5α-DHT/T	*R*	**-0.507**	**-0.481**	-0.386	-0.048	-0.107
	*p*	**0.010**	**0.015**	0.057	0.829	0.628
[αC+ βC+THF]/F	*R*	0.330	**0.413**	0.311	-0.123	0,246
	*p*	0.107	**0.040**	0.131	0.577	0,259
[αCl+ βCl+THE]/E	*R*	-0.396	-0.326	**-0.423**	-0.219	-0.146
	*p*	0.050	0.111	**0.035**	0.316	0.507

Statistically significant values were bolded. BMI, body mass index; BSA, ody Surface Area; CRP, C-reactive protein; FEV_1_, forced expiratory volume in 1 second; Spearman rank correlation coefficients and p values were estimated using Statistica software.

## Discussion

Our study describes for the first time that the urinary excretion of the major glucocorticoid metabolites in CF patients varies from healthy subjects. We have also proven that the activity of the key enzymes involved in the development and metabolism of glucocorticoids differs between the study groups.

The most important finding appears to be altered activity of 11β-hydroxysteroid dehydrogenases (11βHSDs). 11βHSDs are enzymes involved in the balance of steroid hormones. 11β-hydroxysteroid dehydrogenase type 1 functions primarily as a reductase by recovering cortisol from inactive cortisone and has been localized mainly in glucocorticoid-sensitive tissues such as the liver, adipose tissue, testes, central nervous system. 11β-hydroxysteroid dehydrogenase type 2 is expressed in mineralocorticoid-responsive tissues such as the kidney, placenta, colon, salivary, and sweat glands where it rapidly inactivates mainly cortisol to protect tissues from excessive exposure of mineralocorticoid receptors to this hormone (25). The local accumulation of cortisol can cause the apparent mineralocorticoid excess due to activation of mineralocorticoid receptors, lower testosterone levels in male, and fetal developmental defects. Furthermore, this enzyme can also participate in the activation of some androgen precursors (26, 27). It should be noted that glucocorticoids might act in target tissues to increase their own intracellular availability by altering 11βHSD1 activity (28).

Changes in steroid metabolism have been reported in several disorders that affect energy homeostasis, including anorexia nervosa, obesity, and thyroid disorders (29–31). The physiological regulation of short- and long-term fasting poses a great challenge to the human body in maintaining energy balance. The metabolic response to fasting is characterized by a switch from carbohydrate to fat metabolism. During fasting, several adaptive mechanisms are turned on, what is crucial for survival. Insulin secretion is inhibited, while glucagon stimulates glycogenolysis and gluconeogenesis (32). Fasting can also alter the redox state of nicotinamide adenine dinucleotide (NAD), a cofactor involved in many oxidation-reduction enzymatic reactions important for glycolysis, fatty acid oxidation, the Krebs cycle and the function of many enzymes of steroid hormone biosynthesis such as 11βHSD, 3αHSD, SRD5A and SRD5B (25, 33, 34). CF is associated with higher energy consumption, special nutritional deficiencies, and malabsorption primarily related to pancreatic insufficiency (35). There are several reports that have shown differences in the levels of hormones that regulate nutrition, such as leptin, ghrelin, or neuropeptide Y, in CF (36, 37). It was also observed that CF patients had lipid abnormalities, such as a low cholesterol level and a different pattern of lipoproteins. Nowak et al. have reported that hypocholesterolemia occurred in 31% of CF subjects and a lower level of low density lipoprotein (LDL) as well as high density lipoprotein (HDL) (38). Other studies confirm disturbances in lipid levels in CF individuals (39, 40). Our analysis of the lipid profile of CF patients shows that lipoproteins and triglycerides concentrations are low and near the lower limit of the reference values.

We have also revealed that patients with CF show lower excretion of glucocorticoid metabolites measured as single metabolites and as the sum of the main cortisol derivatives (THF+5αTHF+THE, C21 metabolites and cortisol metabolites). Due to the fact that the study group had a wide age and body mass range, we normalized the results by relating them to the BSA of the individuals. 24-hour free urinary cortisol excretion corresponds to plasma free cortisol concentration throughout this period and correction with BSA yielded relatively constant, age- and Tanner stages of sexual development-independent cortisol values in a group of healthy children between 2-17 years of age (24, 41). BSA correction is more useful to normalize excretion of urinary metabolites in children than urinary creatinine correction, because muscle mass, and therefore daily excretion of creatinine, show a physiologically more pronounced increase during growth and/or energy intake than BSA (42–44).

CF is characterized by chronic inflammation primarily of the respiratory system. This may suggest that the levels of cortisol and its metabolites should be elevated, but under physiological conditions, the role of endogenous glucocorticoids is not simply anti-inflammatory or immunosuppressive, but seems to be more immunomodulatory (45). We have found only one study that compared circulating cortisol levels in CF patients and a healthy control. The results were similar to ours and showed lower cortisol levels in the CF group (46). A recent study in young adult women with anorexia nervosa found that chronic starvation led to significant reductions in urinary cortisol (F) and total androgen metabolites compared to healthy controls, and these changes were reversible after refeeding (47). On the other hand, other studies have revealed that the cortisol excretion rate and circadian rhythm are normal in subjects with anorexia nervosa, but the metabolic clearance rate is decreased, leading to hypercortisolemia (48, 49). In women with anorexia nervosa, a decrease of 11βHSD1 activity for the regeneration of active glucocorticoids was also observed (50). A recent study has shown that macrophage 11βHSD1 activity is specifically intense in a chronic inflammatory process such as nonalcoholic fatty liver disease (51). In our study 11βHSD1 activity was significantly increased in CF individuals compared to control whereas 11HSD2 activity seems to be decreased. This may suggest the existence of peripheral compensatory mechanisms that increase cortisol concentrations in target tissues in the context of limited glucocorticoids production by the adrenal glands. It is important to remember that the activities of 11βHSD1 and 11βHSD2 should be evaluated as bidirectional shuttle keeping balance between F and E in an organism. They are also enzymes with different locations in the body. 11βHSD1 seems to maintain proper cortisol availability locally in selected tissues and organs according to this enzyme expression. An unambiguous assessment of 11βHSD2 activity in CF is more challenging, due to the inconclusive results that we have obtained. As mentioned above 11βHSD2 protects mineralocorticoid receptors against their unwanted (with the exception of stress) interaction with cortisol. So, the E/F ratio reflects the concentration of these unmetabolized steroids in the kidneys (probably also in the colon, sweat glands, etc.). On the other hand, the cortolons/cortols ratio mirrors not only 11βHSD2 activity but also their earlier liver metabolism (5β-reductase and 3αHSD complex), which is also changed in CF, and maybe further effects of 20αHSD and 20βHSD activities.

We also found a statistically significant decrease in SRD5A activity. 5α-reductases are a family of three isozymes expressed in many organs and tissues, widely known for converting testosterone into 5α-dihydrotestosterone. It may also have an impact on other steroids, including several androgen and C21 steroids such as corticosterone or cortisol (52). Therefore, SRD5A may reduce the local availability of glucocorticoid, hence its ability to bind and activate the glucocorticoid receptor. The role of 5α-reductase type 2 (SRD5A2) is well known. It contributes to the conversion of testosterone to the most potent androgen 5αDHT, as mentioned above, and is expressed in the testes, prostate, and genital skin. The role of 5α-reductase type 1 (SRD5A1) is defined worse. It is responsible for the conversion of androstenedione to androsterone and probably for the degradation of circulating C21 steroids in the liver in preparation for urinary excretion. It occurs mainly in the skin, brain, and to a lesser extent in the prostate gland (25). Additionally, both type 1 and type 2 isoenzymes can be found in the liver. 5α-reductase type 3 (SRD5A3) is probably involved in the N-glycosylation process and appears ubiquitously expressed in human tissues (53). 5α-reductase often cooperates in duet with 5β-reductase: both enzymes represent a convergence in evolution: they have similar biological functions, but do not share a common ancestor (54). We have found that CF patients show significantly reduced SRD5A activity as measured by ratios of 5α-DHT/T, 5αTHB/THB, 5αTHF/THF. These results have also revealed diminished activity of SRD5A in the degradation of mineralocorticoids (5αTHB/THB) and glucocorticoids (5αTHF/THF). Our findings were confirmed by the An/Et ratio, but the difference was not statistically significant. The 5αTHF/THF ratio can also be used to assess the net balance between 5α and 5β reductase activity. An increase in this ratio may indicate an increase in 5α-reductase or a decrease in a 5β-reductase activity (27). Excretion of cortisol 5α -metabolites is decreased in CF and results in a low urinary 5αTHF/THF ratio, suggesting an additional defect in 5α-reductase activity. Similarly to our results, SRD5A activity was also found to be reduced in patients with anorexia nervosa taking into consideration 5αTHF/THF ratio and critical illness (55, 56). Higher 5α-reductase activity was observed in obese children with insulin resistance, PCOS, and nonalcoholic fatty liver disease (57–59). The conversion ability of SRD5A is probably regulated by androgens, which might increase its activity and thyroid hormones (60). CF is often associated with thyroid insufficiency. Similarly to our results, it has been proven that the 5αTHF/THF and An/Et ratios are significantly lower in hypothyroid patients compared to normal subjects, which could be explained by metabolic adaptation that lowers the resting energy expenditure during the course of the disease (61, 62).

However, 5α-reductase activity seemed to be increased when looking at its activity within the backdoor pathway (11β-OH-An/11β-OH-Et) and after the second crossing of androgen through the adrenal cortex. The backdoor pathway is an alternative biosynthetic route that leads to the production of 5α-DHT without going through testosterone. This pathway seems to be important in male sexual development (63). In the production of 5α-DHT by the backdoor pathway, an important role plays 3α-hydroxysteroid dehydrogenase (3αHSD) that participates only in the backdoor, not in the classic pathway (64). Furthermore, statistical analysis of the ROC curve identified the 11β-OH-An/11β-OH-Et ratio as the best factor for differentiating enzyme activities between study groups. Our results might suggest, enhanced stimulation of the backdoor pathway in CF. These findings were not confirmed with respect to the An/Et ratio, which is a well-established indicator of the backdoor pathway activity (65). It might be explained because crude evaluations of enzymatic activities based only on products/substrates ratios are often difficult and need to consider enzyme location, an intensity of substrate supply, and other clinical circumstances. Not all of these data are possible to recognize precisely enough.

Looking at the activity of 5β-reductase and 3αHSD complex in patients with CF, it seems to be more intense for cortisol metabolism compared to controls and the opposite situation is for cortisone as a starting substrate.

Finally, we evaluated the activity of 20α-hydroxysteroid dehydrogenase (20αHSD) and 20β-hydroxysteroid dehydrogenase (20βHSD), but the results were not statistically significant. The detailed biological roles of these enzymes remain unclear. These enzymes are involved in the final phase of glucocorticoid metabolism that comprises the reduction of tetrahydrocortisol and 5α-tetrahydrocortisol to cortols and tetrahydrocortisone to cortolones. Recent studies have proven that especially 20βHSD is the multispecificity enzyme that plays an important enzyme in stress response because, together with 11βHSD2 rapidly inactivates cortisol (66, 67). 20αHSD and 20βHSD may play a role in the development of PCOS in women due to significant differences in the activities of these enzymes compared to the control (16, 67).

The correlation analysis revealed that 11βHSD1 is positively correlated with age, BMI, and BSA, that is consistent with previous results. 5α-reductase activity is negatively correlated with age and BMI, similar to earlier reports (22). However, our findings did not show a correlation between the spirometry results as well as the inflammatory markers (CRP, IL-6) and the activity of steroid enzymes, which may indicate that lung function and inflammation state do not affect steroids metabolism in the course of CF.

There are some limitations of our study. This was only single center study and the number of the affected participants was low (N= 25) and the age range of the study group was quite wide. We did not measure FEV1, insulin, CRP, and other biochemical parameters in control, so we could miss some findings. In addition, the hormones studied in urine are the final picture of their content, which is an average reflection of many effects such as: the secretory capacity of the adrenal glands, the contribution of other glands producing steroid hormones (gonads), the metabolic ability of individual organs (liver, kidneys, adrenal glands), the influence of proinflammatory cytokines or sequestration in adipose tissue. For this reason, an unambiguous assessment of the activity of enzymes involved in steroid formation and metabolism is difficult. Due to the conclusions of the study should be considered mainly as hypotheses that need to be confirmed in further research. It should be mentioned that glucocorticoids strongly reduce *CFTR* expression and activity, but the molecular effect of cortisol and its metabolites on *CTFR* activity was not the focus of this study.

In conclusion, we have shown a general decrease in the activity of enzymes involved in the peripheral metabolism of cortisol in the liver and other tissues such as 11βHSD2, SRD5A and SRD5B. On the contrary, the activity of 11βHSD1-enzyme responsible for the conversion of the inactive form of glucocorticoid- cortisone to the active agent- cortisol was increased. Furthermore, our study found a significant decrease in glucocorticoid excretion in patients with CF. This may suggest limitations in adrenocortical secretion or dysregulation of the HPA axis, of which we cannot fully explain the cause. Hormone dysregulation might be another important factor in the course of CF that should be taken into account when planning an effective and comprehensive therapy. The regulation mechanism of steroid hormone metabolism is very complex and is not well understood, especially in CF, so further studies are required to explain in detail the role of enzymes involved in this process, as well as the plausible impact of pro-inflammatory cytokines and oxidative stress agents that can change intracellular concentrations of bioactive glucocorticoids.

## Data availability statement

The raw data supporting the conclusions of this article will be made available by the authors, without undue reservation.

## Ethics statement

The studies involving human participants were reviewed and approved by Bioethical Committee of the University of Rzeszow. Written informed consent to participate in this study was provided by the participants’ legal guardian/next of kin.

## Author contributions

RP, MR, and AM: Conceptualization and study design; MR, MS and PF: Patient care and samples gathering; RP: Data acquisition; RP and PF: Data analysis; RP, PF, MS: Writing - original draft; MF, MR, AM: Writing - review and editing manuscript. All authors contributed to the article and approved the submitted version.
