# Supplementary material for: Alteration in glucocorticoids secretion and metabolism in patients affected by cystic fibrosis
Source: Front Endocrinol (Lausanne). 2022 Dec 8;13:1074209. doi: 10.3389/fendo.2022.1074209 (PMC9779927; doi:10.3389/fendo.2022.1074209)
Supplement: Supplementary file 1 [file DataSheet_1.docx]

**Quantification of Urinary Steroid Metabolites and instrument conditions**

**1. Materials**

Standards, reagents, and enzymes were obtained as follows: All steroid standards including medroxyprogesterone, cholesteryl butyrate, and stigmasterol were obtained from Steraloids (Newport RI, USA), the Lipidex 5000 from Perkin Elmer (Waltham MA, USA), the Sep-Pak C18 column from Waters (Warsaw, Poland). Other chemicals as: the β-glucuronidase/arylsulfatase, the sulfatase type H-1 from Helix pomatia, derivative agents methoxyamine hydrochloride and trimethylsilylimidazole (TMSI), pyridine, sodium acetate and acetic acid from Sigma–Aldrich (Poznan, Poland).

2. Method

The method involves a 3-day sample preparation that includes initial extraction of a urine sample (1,5 ml) on a Sep-Pak C18 cartridge with the recovery standard medroxyprogesterone, enzymatic hydrolysis, extraction of the unconjugated steroids again on a Sep-Pak column, derivatization with methoxyamine hydrochloride 2% in pyridine at 60˚C for 3 hours and next with TMSI at 100˚C for 16 hours after adding the two internal standards Stigmasterol and Cholesteryl butyrate purification on a Lipidex 5000 column. Finally, the dried residue was resuspended in cyclohexane and analyzed by GC-MS in selected ion monitoring mode (SIM).

GC-MS was performed with a Shimadzu 2010 Plus gas chromatograph (Kyoto, Japan) interfaced with a single-quadrupole Shimadzu QP-2010 Ultra mass spectrometer at an electron energy of 70 eV, ion source temperature 230°C and interface temperature 250°C. Each sample (1 µl) was injected in splitless mode at 260°C using the Shimadzu AOC-20i auto injector and separated through a ZB-1ms capillary column (15 m × 0.25 mm I.D., 0.25 µm film thickness -Pheanomenex, Torrance, USA).

The GC oven temperature was initially set at 50 °C which was held for 3 minutes, then increased to 210°C at 30°C/min, next to 265°C at 2°C/min and finally increased to 320°C using a 20°C/min ramping program over a period of 48-min period. The carrier gas was helium in the linear velocity flow control mode (column flow 1,2 ml/min.). For quantitative analysis, the specific ions of each steroid metabolites were determined as their MO-TMS derivatives (Table S1).

The instrument was calibrated by analysing standard mixtures containing known amounts of reference steroids and internal standards. The area of the obtained peaks was measured in SIM mode, and a six-level calibration curve was set for each analyte. The recoveries were checked with medroxyprogesterone and corrections were made for losses that occurred during sample preparation. Method validation information for all quantified steroids has previously been described (18). For all measured steroids, specific peaks in the chromatograms had to be three times higher as the noise above the baseline to be counted as valid measurements (signal-to-noise-ratio 3). Urine samples from the same healthy volunteer were measured in all measurement series and the results are compared with the standard values derived from 10 measurements of this volunteer. For quality control, the quantitative results must be within ±30% of the individual reference intervals.

**Table S1.** List of steroid compounds measured in urine.

| **Trivial name** | **Abbreviation** | **Systematic name** | **M** | **RT** | **QIon** | **Ref. Ion** |
| --- | --- | --- | --- | --- | --- | --- |
| Androsterone | An | 5α-androstan-3α-ol-17-one | 290.4 | 13.5 | 270.2 | 360.2 |
| Etiocholanolone | Et | 5β-androstan-3α-ol-17-one | 290.4 | 13.7 | 270.2 | 360.2 |
| 11β-Hydroxyandrosterone | 11β-OH-An | 5α-androstan-3α, 11β-diol-17-one | 306.4 | 16.4 | 268.2 | 448.4 |
| 11β-Hydroxyetiocholanolone | 11β-OH-Et | 5β-androstan-3α, 11β-diol-17-one | 306.4 | 16.7 | 268.2 | 448.4 |
| 5α-Dihydrotestosterone | 5α-DHT | 5α-androstan-17β-ol-3-one | 290.4 | 15.2 | 391.3 | 360.2 |
| Testosterone | T | 4-androsten-17β-ol-3-one | 288.4 | 15.6 | 389.3 | 268.2 |
| Tetrahydrocorticosterone | THB | 5β-pregnan-3α, 11β, 21-triol-20-one | 350.5 | 22.7 | 564.5 | 474.4 |
| 5α-Tetrahydrocorticosterone | 5α-THB | 5α-pregnan-3α, 11β, 21-triol-20-one | 350.5 | 23.1 | 564.5 | 472.3 |
| Tetrahydrocortisone | THE | 5β-pregnan-3α, 17, 21-triol-11, 20-dione | 364.5 | 21.9 | 578.5 | 488.4 |
| Tetrahydrocortisol | THF | 5β-pregnan-3α, 11β, 17, 21-tetrol-20-one | 366.5 | 23.3 | 652.6 | 472.3 |
| 5a-Tetrahydrocortisol | 5α-THF | 5α-pregnan-3α, 11β, 17, 21-tetrol-20-one | 366.5 | 23.6 | 652.6 | 551.4 |
| α -Cortolone | αCl | 5β-pregnan-3α, 17, 20α, 21-tetrol-11-one | 366.5 | 24.0 | 449.4 | 523.4 |
| β-Cortol | βC | 5β-pregnan-3α, 11β, 17, 20β, 21-pentol | 368.5 | 24.7 | 343.3 | 551.4 |
| β-Cortolone | βCl | 5β-pregnan-3α, 17, 20β, 21-tetrol-11-one | 366.5 | 24.8 | 449.4 | 253.4 |
| α -Cortol | αC | 5β-pregnan-3α, 11β, 17, 20α, 21-pentol | 368.5 | 25.7 | 343.3 | 562.5 |
| Cortisone | E | 4-pregnen-17, 21-diol-3, 11, 20-trione | 360.5 | 27.2 | 531.5 | 515.4 |
| Cortisol | F | 4-pregnen-11β, 17, 21-triol-3, 20-dione | 362.5 | 29.2 | 605.5 | 488.4 |
| 20β-Dihydrocortisone | 20β-DHE | 4-pregnen-17, 20β, 21-triol-3, 11-dione | 362.5 | 29.3 | 402.4 | 488.4 |
| 20α -Dihydrocortisone | 20α-DHE | 4-pregnen-17, 20α, 21-triol-3, 11-dione | 362.5 | 30.0 | 402.4 | 476.3 |
| 20β-Dihydrocortisol | 20β-DHF | 4-pregnen-11β, 17, 20β, 21-tetrol-3-one | 364.5 | 30.1 | 296.2 | 513.5 |
| 6β-hydroxycortisol | 6β-OH-F | 4-pregnen-6β, 11β, 17, 21-tetrol-3, 20-dione | 378.5 | 30.6 | 693.4 | 476.3 |
| 20α -Dihydrocortisol | 20α -DHF | 4-pregnen-11β, 17, 20α, 21-tetrol-3-one | 364.5 | 31.2 | 296.2 | 490.4 |
| Stigmasterol | SS | 5, 22-cholestadien-24β-ethyl-3β-ol | 412.7 | 28.3 | 394.3 | 353.2 |
| Cholesterol N-butyrate | CB | 5-cholesten-3β-ol n-butyrate | 456.8 | 31.9 | 368.2 | 360.2 |
| Medroxyprogesteron | MP | 4-pregnen-6α-methyl-17-ol-3, 20-dione | 344.5 | 21.8 | 443.4 | 400.3 |

Abbreviations list: M-molar mass [g/mol], RT- retention time [min], QIon: quantifier ion [m/z], Ref. Ion- reference ion [m/z]
