# Supplementary material for: Alteration in glucocorticoids secretion and metabolism in patients affected by cystic fibrosis
Source: Front Endocrinol (Lausanne). 2022 Dec 8;13:1074209. doi: 10.3389/fendo.2022.1074209 (PMC9779927; doi:10.3389/fendo.2022.1074209)
Supplement: Supplementary file 2 [file DataSheet_2.docx]

Figure S1. Results of the analysis of the ROC curve for (THF+5αTHF)/(THE) ratio. The shaped blue area is the 95% confidence interval of the sensitivity at the given specificity.


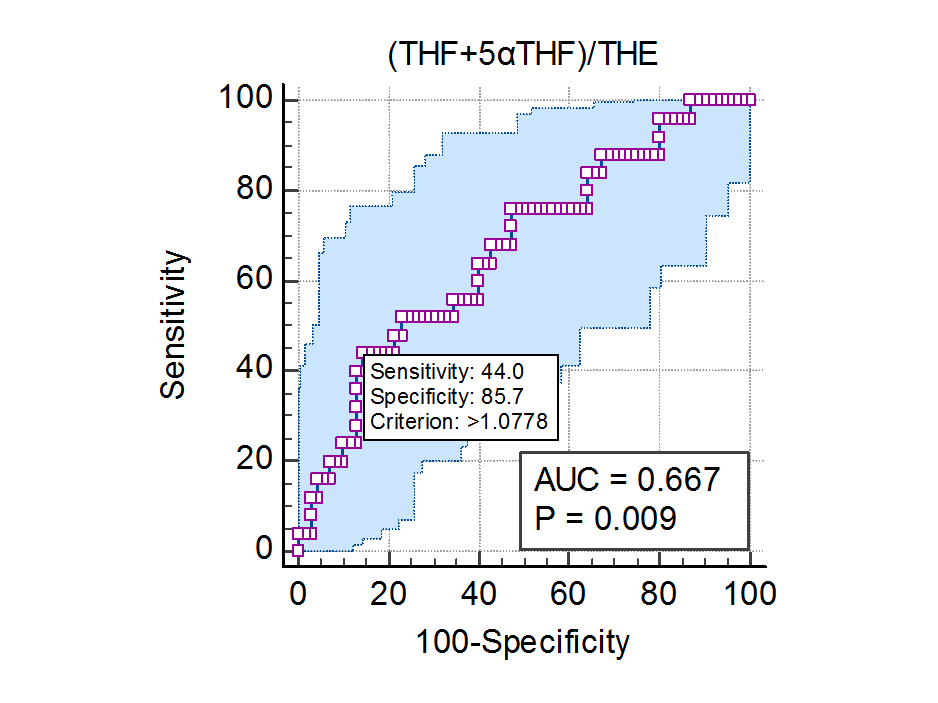


**Area under the ROC curve (AUC)**

| Area under the ROC curve (AUC) | 0.667 |
| --- | --- |
| Standard Error ^a^ | 0.0638 |
| 95% Confidence interval ^b^ | 0.563 to 0.761 |
| z statistic | 2.626 |
| Significance level P (Area=0.5) | 0.0086 |

^a^ DeLong et al., 1988

^b^ Binomial exact

**Youden index**

| Youden index J | 0.2971 |
| --- | --- |
| Associated criterion | >1.077771141 |
| Sensitivity | 44.00 |
| Specificity | 85.71 |

Figure S2. Results of the analysis of the ROC curve for E/F ratio. The shaped blue area is the 95% confidence interval of the sensitivity at the given specificity.

**Area under the ROC curve (AUC)**

| Area under the ROC curve (AUC) | 0.678 |
| --- | --- |
| Standard Error ^a^ | 0.0686 |
| 95% Confidence interval ^b^ | 0.574 to 0.770 |
| z statistic | 2.590 |
| Significance level P (Area=0.5) | 0.0096 |

^a^ DeLong et al., 1988

^b^ Binomial exact

**Youden index**

| Youden index J | 0.3600 |
| --- | --- |
| Associated criterion | >0.961438068 |
| Sensitivity | 76.00 |
| Specificity | 60.00 |

**Criterion values and coordinates of the ROC curve** [[Show]](javascript:showdiv('d6','d7','table1');)

Figure S3. Results of the analysis of the ROC curve for cortolones/cortols ratio. The shaped blue area is the 95% confidence interval of the sensitivity at the given specificity.

**Criterion values and coordinates of the ROC curve** [[Show]](javascript:showdiv('d10','d11','table1');)

**Area under the ROC curve (AUC)**

| Area under the ROC curve (AUC) | 0.727 |
| --- | --- |
| Standard Error ^a^ | 0.0610 |
| 95% Confidence interval ^b^ | 0.626 to 0.813 |
| z statistic | 3.716 |
| Significance level P (Area=0.5) | 0.0002 |

^a^ DeLong et al., 1988

^b^ Binomial exact

**Youden index**

| Youden index J | 0.4343 |
| --- | --- |
| Associated criterion | ≤2.437046579 |
| Sensitivity | 72.00 |
| Specificity | 71.43 |

Figure S4. Results of the analysis of the ROC curve for An/Et ratio. The shaped blue area is the 95% confidence interval of the sensitivity at the given specificity.

**Area under the ROC curve (AUC)**

| Area under the ROC curve (AUC) | 0.538 |
| --- | --- |
| Standard Error ^a^ | 0.0661 |
| 95% Confidence interval ^b^ | 0.433 to 0.641 |
| z statistic | 0.579 |
| Significance level P (Area=0.5) | 0.5627 |

^a^ DeLong et al., 1988

^b^ Binomial exact

**Youden index**

| Youden index J | 0.1543 |
| --- | --- |
| Associated criterion | ≤2.373938896 |
| Sensitivity | 84.00 |
| Specificity | 31.43 |

Figure S5. Results of the analysis of the ROC curve for 11β-OH-An/11β-OH-Et ratio. The shaped blue area is the 95% confidence interval of the sensitivity at the given specificity.

**Area under the ROC curve (AUC)**

| Area under the ROC curve (AUC) | 0.846 |
| --- | --- |
| Standard Error ^a^ | 0.0468 |
| 95% Confidence interval ^b^ | 0.758 to 0.912 |
| z statistic | 7.405 |
| Significance level P (Area=0.5) | <0.0001 |

^a^ DeLong et al., 1988

^b^ Binomial exact

**Youden index**

| Youden index J | 0.7057 |
| --- | --- |
| Associated criterion | >9.402269511 |
| Sensitivity | 92.00 |
| Specificity | 78.57 |

Figure S6. Results of the analysis of the ROC curve for 5α-DHT/T. The shaped blue area is the 95% confidence interval of the sensitivity at the given specificity.

**Area under the ROC curve (AUC)**

| Area under the ROC curve (AUC) | 0.646 |
| --- | --- |
| Standard Error ^a^ | 0.0762 |
| 95% Confidence interval ^b^ | 0.541 to 0.741 |
| z statistic | 1.913 |
| Significance level P (Area=0.5) | 0.0558 |

^a^ DeLong et al., 1988

^b^ Binomial exact

**Youden index**

| Youden index J | 0.3914 |
| --- | --- |
| Associated criterion | ≤0.280333156 |
| Sensitivity | 52.00 |
| Specificity | 87.14 |

Figure S7. Results of the analysis of the ROC curve for 5α-THB/THB ratio. The shaped blue area is the 95% confidence interval of the sensitivity at the given specificity.

**Area under the ROC curve (AUC)**

| Area under the ROC curve (AUC) | 0.687 |
| --- | --- |
| Standard Error ^a^ | 0.0601 |
| 95% Confidence interval ^b^ | 0.585 to 0.778 |
| z statistic | 3.115 |
| Significance level P (Area=0.5) | 0.0018 |

^a^ DeLong et al., 1988

^b^ Binomial exact

**Youden index**

| Youden index J | 0.3425 |
| --- | --- |
| Associated criterion | >0.219661218 |
| Sensitivity | 92.00 |
| Specificity | 42.25 |

Figure S8. Results of the analysis of the ROC curve for 5α-THF/THF ratio. The shaped blue area is the 95% confidence interval of the sensitivity at the given specificity.

## Area under the ROC curve (AUC)

| Area under the ROC curve (AUC) | 0.688 |
| --- | --- |
| Standard Error ^a^ | 0.0578 |
| 95% Confidence interval ^b^ | 0.586 to 0.779 |
| z statistic | 3.259 |
| Significance level P (Area=0.5) | 0.0011 |

^a^ DeLong et al., 1988

^b^ Binomial exact

## Youden index

| Youden index J | 0.3420 |
| --- | --- |
| Associated criterion | >0.771610923 |
| Sensitivity | 68.00 |
| Specificity | 66.20 |

## Criterion values and coordinates of the ROC curve [[Show]](javascript:showdiv('d2','d3','table1');)

**Criterion values and coordinates of the ROC curve** [[Show]](javascript:showdiv('d0','d1','table1');)

Figure S9. Results of the analysis of the ROC curve for [αC+ βC+THF]/F ratio. The shaped blue area is the 95% confidence interval of the sensitivity at the given specificity.

**Area under the ROC curve (AUC)**

| Area under the ROC curve (AUC) | 0.685 |
| --- | --- |
| Standard Error ^a^ | 0.0638 |
| 95% Confidence interval ^b^ | 0.582 to 0.777 |
| z statistic | 2.902 |
| Significance level P (Area=0.5) | 0.0037 |

^a^ DeLong et al., 1988

^b^ Binomial exact

**Youden index**

| Youden index J | 0.3029 |
| --- | --- |
| Associated criterion | >15.391331547 |
| Sensitivity | 36.00 |
| Specificity | 94.29 |

Figure S10. Results of the analysis of the ROC curve for [αCl+ βCl+THE]/E ratio. The shaped blue area is the 95% confidence interval of the sensitivity at the given specificity.

**Area under the ROC curve (AUC)**

| Area under the ROC curve (AUC) | 0.721 |
| --- | --- |
| Standard Error ^a^ | 0.0679 |
| 95% Confidence interval ^b^ | 0.619 to 0.808 |
| z statistic | 3.248 |
| Significance level P (Area=0.5) | 0.0012 |

^a^ DeLong et al., 1988

^b^ Binomial exact

**Youden index**

| Youden index J | 0.4000 |
| --- | --- |
| Associated criterion | ≤33.894616355 |
| Sensitivity | 80.00 |
| Specificity | 60.00 |

Figure S11. Results of the analysis of the ROC curve for [αC+αCl]/[THF+5αTHF+THE] ratio. The shaped blue area is the 95% confidence interval of the sensitivity at the given specificity.

**Area under the ROC curve (AUC)**

| Area under the ROC curve (AUC) | 0.620 |
| --- | --- |
| Standard Error ^a^ | 0.0693 |
| 95% Confidence interval ^b^ | 0.515 to 0.718 |
| z statistic | 1.731 |
| Significance level P (Area=0.5) | 0.0834 |

^a^ DeLong et al., 1988

^b^ Binomial exact

**Youden index**

| Youden index J | 0.2286 |
| --- | --- |
| Associated criterion | ≤0.249247965 |
| Sensitivity | 60.00 |
| Specificity | 62.86 |

Figure S12. Results of the analysis of the ROC curve for [βC+βCl]/[THF+5αTHF+THE] ratio. The shaped blue area is the 95% confidence interval of the sensitivity at the given specificity.

**Area under the ROC curve (AUC)**

| Area under the ROC curve (AUC) | 0.574 |
| --- | --- |
| Standard Error ^a^ | 0.0699 |
| 95% Confidence interval ^b^ | 0.468 to 0.675 |
| z statistic | 1.054 |
| Significance level P (Area=0.5) | 0.2920 |

^a^ DeLong et al., 1988

^b^ Binomial exact

**Youden index**

| Youden index J | 0.1943 |
| --- | --- |
| Associated criterion | ≤0.1434364 |
| Sensitivity | 48.00 |
| Specificity | 71.43 |
